# An Instrument to Operationalize the Balance between Risks and Resources and Predict Job Burnout

**DOI:** 10.3390/ijerph18179416

**Published:** 2021-09-06

**Authors:** Neda Bebiroglu, Marie Bayot, Benjamin Brion, Léopold Denis, Thomas Pirsoul, Isabelle Roskam, Moïra Mikolajczak

**Affiliations:** 1 Psychological Sciences Research Institute, Université Catholique de Louvain, 1348 Louvain-la-Neuve, Belgium; neda.bebiroglu@uclouvain.be (N.B.); thomas.pirsoul@uclouvain.be (T.P.); isabelle.roskam@uclouvain.be (I.R.); moira.mikolajczak@uclouvain.be (M.M.); 2 Observatory of Research and Scientific Careers—F.R.S.-FNRS, 1000 Brussels, Belgium; 3 Department of Clinical Sciences, Université de Liège, 4000 Liège, Belgium; 4 Research and Development, Moodwalk, 2 bis rue Vermenton, 60 200 Compienge, France; benjamin@moodwork.com (B.B.); leopold@moodwork.com (L.D.)

**Keywords:** job demands, job resources, job satisfaction, job turnover intention, counterproductive behaviors at work, health

## Abstract

The goal of the present paper was to develop a valid and reliable instrument to operationalize the balance between job demands and resources in order to predict job burnout. After generating the items, we first conducted a cross-sectional study (Study 1) based on 656 participants, which provided preliminary evidence for the validity of the balance. We then conducted a longitudinal study (Study 2) based on 882 participants to improve and validate the final version of the balance. In study 1, the (im)balance between risks and resources explained a high percentage of variance in job burnout (44%) and a significant percentage in job turnover intention (27%) as well as subjective health (12%). In study 2, results indicated that a change in the balance produced significant change in job burnout scores over time. In addition, balance scores positively predicted positive outcomes (i.e., overall job satisfaction and subjective health) and negatively predicted negative outcomes (i.e., job turnover intention, counterproductive behaviors at work, depression, alcohol use, sleep disorders and somatic complaints). Findings support the usefulness of the Balance for clinicians, companies and researchers interested in assessing job demands and resources.

## 1. Introduction

Since it has emerged in the 1970s, the phenomenon of burnout has received ever increasing attention both inside and outside academia. Job burnout is a specific disorder resulting from prolonged exposure to high job demands in the absence of enough resources to compensate for their effects [1,2]. The prevalence of job burnout greatly varies across occupational sectors and countries (and according to the cut-offs used) but it is generally accepted that approximately 15% of workers will experience job burnout [3,4]. This is preoccupying as a considerable amount of research has linked burnout to deleterious outcomes for both the employee and the organization. For instance, burnout is associated with physical health impairments [5,6,7,8,9,10], medication use [11], and sleep disturbances [12,13]. In addition, job burnout has important consequences for organizations: it has been associated with increased intention to leave [14], absenteeism [15], work-place accidents and injuries [16], reduced levels of performance [17] and decreased organizational commitment [6]. Therefore, there is a crucial need for organizations to identify employees who are at risk to prevent burnout, as well as to intervene with those who already suffer from it.

### 1.1. The Job Demands-Resources Model

The job demands-resources model/theory (the JD-R) [2,15,18,19] presents a parsimonious model to understand which perceived working conditions can predict burnout. According to this model, working conditions can be categorized into two overarching categories: job demands and job resources. Job demands refer to physical, social, or organizational characteristics of a work that require sustained effort from employees and are therefore associated with certain costs. Job demands are stress-increasing factors that increase exhaustion. Work overload or time pressure represent such factors. On the other hand, job resources refer to those aspects of one’s job that contribute toward reducing the effect of job demands and their related costs, are functional in achieving work goals, and stimulate personal development. Job resources can include aspects such as opportunities for development and support from colleagues.

According to this model, two processes can explain how job demands and resources are associated with burnout and/or engagement [20]. The first process, which is driven by job demands, is called the strain process. When faced with increasing job demands, an employee who would like to maintain performance levels engage in compensatory effort to achieve his/her goals. This extra effort comes with physiological and psychological costs such as irritability or fatigue. If this compensatory effort is continuously used, it drains the employee’s energy and may ultimately lead to burnout. The second process, which is driven by job resources, is called the motivational process. Job resources play an intrinsic motivational role in fulfilling basic needs for autonomy, relatedness, and competence as postulated in self-determination theory [21]. For instance, social support from colleagues may satisfy the need for relatedness whereas opportunities for development may increase the need for competence. If an employee perceives his/her needs to be satisfied at work, he/she will be more motivated to work. In addition to their intrinsic motivational role, job resources play an extrinsic motivational role since they increase the willingness to spend compensatory effort [22].

The strain process and motivational process have been supported through empirical evidence both cross-sectionally and longitudinally (see for review [22]). For instance, job resources have been found to influence future work engagement, which in turn has been linked to organizational commitment (motivational process) [23], whereas job demands have been linked to burnout, which in turn has predicted depression (strain process) [20,24]. The strong empirical evidence in favor of the JD-R model has made it the dominant explanatory theory of job burnout. One of its advantages is that the model/theory can accommodate for a varying number of risks and resources.

Although the JD-R model has been widely used as a conceptual framework in multiple studies, risks and resources have always been measured separately. At present, there is no instrument that operationalizes the *balance* between risks and resources. Our primary objective was to develop a valid and conceptually reliable instrument to assess the *balance* between risks and resources based on the JD-R theory.

### 1.2. Operationalizing the Balance between Risks and Resources

To efficiently operationalize the (im)balance between risks and resources, we sought a format that would intrinsically reflect the notion of balance. Therefore, instead of creating a questionnaire that would measure risks and resources separately, as is the case in the literature at present, we developed bipolar items, in which the left pole represents the risk factors (i.e., factors that increase job-related stress) and the right pole the resources (i.e., factors that alleviate job-related stress). For instance, for the item on support from colleagues, the left pole reads “I can never count on my colleagues” and the right pole reads “I can easily rely on my colleagues”. For the item on new technologies, the left pole reads “The use of new technologies complicates my work (e.g., programs become more and more complicated; more and more codes to remember, etc.)” whereas the right pole reads “The use of new technologies greatly facilitates my work (thanks to new technologies, I no longer have to perform tasks that I didn’t like or I save time on certain tasks compared to before, etc.)”. The response scale goes from −5 (full endorsement of the risk factor) to +5 (full endorsement of the resource factor), 0 indicating that the participant has neither the risk nor the resource (in this case: I do not feel particularly supported or not supported by my colleagues; new technologies neither impede nor facilitate my job).

Provided that the questionnaire includes the most important risk/resource factors and that these are appropriately weighted (e.g., heavier risks/protections reflected by more items; see below), the arithmetic sum of the answers to such a questionnaire (this arithmetic sum logically involves subtracting risks from resources) reflects the respondent’s balance between risks and resources (see [25] for the validation of a comparable instrument in the context of parental burnout). If a participant has more or heavier risk factors, the score will be negative; if resources just compensate for risks, the score will be zero; and if a participant has more or heavier resources, the score will be positive. According to the JD-R theory, we would expect that burnout occurs when risk factors outweigh resources factors, i.e., when people’s score at the balance is negative (below zero).

### 1.3. Overview of Studies

The balance between risks and resources (henceforth named “Balance”) was developed using a multi-step method described below. Step 1 consisted in the literature review and the item generation. Step 2 comprised the item confirmation and preliminary validation of the instrument through Study 1 (cross-sectional study, N = 656). Step 3 consisted in the improvement and validation of the final version of the instrument through Study 2 (three-wave longitudinal study; N = 882).

### 1.4. Item Generation

The 35 items that made up the first version of the Balance were generated by experts on burnout and psychometrics, based on a literature review of individual and organizational factors that increase or decrease occupational stress and a review of instruments measuring these factors. When available, meta-analyses were used to obtain a more accurate estimate of the weight of each factor (e.g., [16,26,27,28,29,30]). We included in the Balance all factors that, based on previous studies, had at least a weak association with job stress or burnout (i.e., *r* > |0.2|). Since factors with the strongest association with job burnout (e.g., workload) need to be given more weight, these factors were represented by more items. Three authors gathered together during several hours to produce items. The items were then reviewed by two other authors for clarity. Several items were then reworked until final agreement was reached among authors that the items clearly and unambiguously measured the variables at stake.

## 2. Study 1: Item Confirmation and Validation of the Principle of the Balance

The goal of Study 1 was to get a preliminary idea of the validity of the Balance and to understand how the items generated functioned. More specifically, we aimed to answer the following research questions: (1) how is the Balance related to job burnout, subjective health and turnover intention? (2) how are the items of the Balance related to the job burnout? and (3) how much does the Balance add to the prediction of job burnout above and beyond sociodemographic factors?

### 2.1. Method

#### 2.1.1. Participants

Participation in this study was voluntary and anonymous. All participants provided written consent after receiving information about the study. We included only participants who indicated to have a job. The final sample consisted of 656 participants (64.6% female) located predominantly in French-speaking countries (Belgium 48%, France 48.3%, and other (e.g., Switzerland, 3%). The age of respondents ranged from 20 to 71 years (*M* = 39.28; *SD* = 9.96). 21.5% of participants had a master’s or a doctoral degree, 45.3% of participants had a bachelor’s degree, 23.9% had some college degree or vocational training, and 9.3% had a high school diploma. Of the participants, 3.5% indicated to work between 6 to 20 h per week, 15.1% between 21 to 34 h per week and 81.4% indicated to work 35 h or more.

#### 2.1.2. Procedure

Participants were recruited mostly by word of mouth and social networks. The invitation to participate included a short description of the study, eligibility criteria for participation (i.e., to have a job), and a hyperlink to the survey, which directed participants to Qualtrics, a secure online data collection software. Participants’ data were automatically downloaded into a database for statistical analyses. Study link was activated on November 16th 2016 and closed two weeks after. Sixty-four percent of individuals who logged on the survey page (*N* = 1143) answered all questions.

#### 2.1.3. Measures

In addition to sociodemographic questions (age, country of residence, gender, education, and work status), we measured the balance, job burnout, job turnover intention, and subjective health. We used a forced-choice format in Qualtrics to prevent missing data. Reliability for all measures was estimated using Cronbach’s alpha and is presented in Table 1. All measures had good internal consistency.

*Balance* was measured using 35 bipolar items encompassing 11 points, rated from −5 to +5, including 0. The negative pole represents the risk (e.g., “I feel insufficiently rewarded for my work), and the positive pole the corresponding resource (e.g., “I feel rewarded for my work”). The global score is computed by summing up the items. Positive scores indicate that the respondent has more (or heavier) resources than risks and negative scores indicate that the respondent has more (or heavier) risks than resources. A zero score means that the respondent has the same level of risks and resources. Reliabilities were not computed for this measure since risk and resources are not necessarily expected to covary (i.e., a person who receives high support from colleagues is not necessarily well paid).

*Job burnout* was assessed with the Maslach Burnout Inventory–General Survey (MBI-GS; [31], validated in French [32]. The French version of MBI-GS has similar factorial validity and internal consistency as the original version. The MBI-GS includes three factors: exhaustion (five items; e.g., “I feel emotionally drained from my work”), cynicism (five items; e.g., “I have become less enthusiastic about my work”), and professional efficacy (six items; e.g., “I have accomplished many worthwhile things in this job”). Participants respond how often they feel this way about their job on a 7-point Likert-type scale from 0 = never to 6 = every day. Given that we were not interested in the specific dimensions of burnout, we computed a global score of burnout after reversing the items for professional efficacy. Higher scores indicating higher levels of burnout.

*Job turnover intention* was assessed by averaging two items: “I often think about quitting my company”, “I am actively looking for a position with another employer” [33]. Respondents indicated their level of agreement with each item using a 7-point Likert scale (0 = never or less than once a year to 6 = a few times a day).

*Subjective health* was assessed by averaging four items indicating how much participants considered themselves in good physical health using a 7-point Likert scale [34]. Participants either self-rated themselves (e.g., “In general I consider myself” (responses ranged from 1 = not a very healthy person to 7 = a very healthy person) or compared themselves to others (e.g., “Compared with most of my acquaintances, I consider myself” (responses ranged from 1 = less healthy or 7 = much healthier).

#### 2.1.4. Data Analysis

Preliminary analyses involved computing means and reliabilities of each variable. To address the first research question, we computed linear correlations among variables of interest. To address the second research question, we computed linear correlations between each item of the Balance and job burnout, thereby yielding specific weight indices of each stress-inducing factor in predicting the syndrome. To further probe the relationship between risk and resources and job burnout and address the third research question, we used a linear regression model. In Step 1, we entered gender, age, and work status (Full time versus part-time or less) as sociodemographic control factors (Model 1). In Step 2 we added the Balance score, in order to assess its effect above and beyond sociodemographic factors (Model 2).

### 2.2. Results

Table 1 shows the means, standard deviation, possible range, reliability estimates and intercorrelations of the risk and resources, job burnout, job turnover intention, and subjective health. Correlation results indicated direct and significant relationships between the Balance and all variables of interest. We found a negative relation between the Balance and the MBI and between the Balance and job turnover intention. As predicted by the JD-R theory, participants whose balance leaned to the positive side had significantly lower burnout scores (*r* = −0.66, *p* < 0.001) and had significantly lower job turnover intention (*r* = −0.52, *p* < *0*.001); they also reported higher subjective health (*r* = 0.35, *p* < *0*.001). Table 2 presents the correlations between the items of the Balance and the MBI. All items of the Balance were negatively correlated with the MBI, except for item number 3 on workload.

The linear regression model showed a significant contribution of sociodemographic factors to the prediction of job burnout (*R*^2^ = 0.03, *F* (3, 652) = 6.34, *p* < 0.001). This model (Model 1) accounted for 3% of the variance in job burnout scores. Females and younger participants reported higher job burnout scores. The model supplemented with the Balance score (Model 2) accounted for a very significant amount of variance in job burnout in comparison to Model 1 (*ΔR*^2^ = 0.47; *R*^2^ = 0.50, *F* (4, 651) = 162.13, *p* < 0.001). After accounting for the three control variables, participants whose balance leaned to the negative side reported higher burnout scores (β = −0.69, *p* < 0.001). For a full summary, see Table 3.

### 2.3. Brief Discussion

Study 1 provided preliminary evidence for the validity of the Balance. As predicted by the JD-R theory, the (im)balance between risks and resources explained a high percentage of variance in job burnout. The Balance also significantly predicted job turnover intention as well as subjective health. Regarding the items specifically, we found support for the validity of 34 items. The study showed, however, that the notion of workload was not accurately captured by the current version of the Balance: Workload did not correlate in the expected direction with job burnout and other criteria, nor did it have the predicting weight that it should have based on previous studies. One of the goals of Study 2 was to fix this weakness.

## 3. Study 2: Improvement and Validation of the Final Version of the Balance

Study 2 aimed to improve the Balance based on the results of Study 1 and examine the validity of the resulting instrument using a stronger methodology. We improved the Balance instrument in two ways: first, we generated four new items in order to give workload the weight that it should have according to previous studies. Second, we added an item to capture meaning at work. We also improved the study design in two ways: first, we used a three-wave longitudinal design. Second, we included a greater number of criterion variables, i.e., job burnout, overall job satisfaction, job turnover intention, counterproductive behaviors at work, depression, problematic alcohol use, disordered sleep and somatic complaints. We thus examined two research questions: (1) how is the balance between risks and resources related to job burnout, subjective health, turnover intention, job satisfaction, counterproductive behaviors at work, problematic alcohol use, disordered sleep and somatic complaints over time? and (2) how does intra-individual changes in the Balance account for intra-individual changes in burnout over time?

### 3.1. Method

#### 3.1.1. Participants

At Time 1, the sample consisted of 882 participants (58.7% female) located in the United Kingdom (65.8%), the United States (25.1%) and other countries 9.1% (e.g., Canada). The age of respondents ranged from 20 to 63 years (*M* = 38.24; *SD* = 8.21). 18.2% of participants had a master’s or a doctoral degree, 43% of participants had a bachelor’s degree, 37.2% had a high school diploma and 1.7% had an elementary school diploma. 73.5% indicated to work full-time and 26.5 indicated to work part-time.

At Time 2 (Time 1 + 4 months), the sample consisted of 558 participants (57.3% female) located in the United Kingdom (66.9%), the United States (22.4%) and other countries (10.7%). The age of respondents ranged from 21 to 63 years (*M* = 39.34; *SD* = 7.86). 18.1% of participants had a master’s or a doctoral degree, 45.2% of participants had a bachelor’s degree, 35.5% had a high school diploma and 1.3% had an elementary school diploma. 74% indicated to work full-time and 26% indicated to work part-time.

At time 3 (Time 1 + 8 months), the sample consisted of 509 participants (56.4% female) located mostly in the United Kingdom (64.5%), the United States (23.4%) and other countries (11.1%). The age of respondents ranged from 21 to 63 years (*M* = 39.49; *SD* = 7.69). 18.4% of participants had a master’s or a doctoral degree, 44.2% of participants had a bachelor’s degree, 35.9% had a high school diploma and 1.4% had an elementary school diploma. 74.1% indicated to work full-time, and 25.9% indicated to work part-time.

As a dropout was observed between the waves, we conducted an analysis of missingness. Following the recommended steps [35], we first examined the missing values in each wave through logistic regression. Predictors of missingness (demographic variables), i.e., age, gender, work status, education level, were entered in logistic regressions with the binary drop-out in each wave as the dependent variable. We found differential attrition for age from Wave 1 to Wave 2 (*B* = 0.04, *p* < 0.001) and to Wave 3 (*B* = 0.04, *p* < 0.001). Based on these results, age was controlled in following analyses.

#### 3.1.2. Procedure

This study was approved by the Ethical Committee. Participation in this study was voluntary (all participants provided informed consent) and anonymous. Participants were recruited from Prolific Academic, an online crowdsourcing platform, which has been shown to produce higher data quality compared to other platforms such as Amazon’s Mechanical Turk [36]. All members of the platform could see the study announcement, but only participants who had a job were eligible to fill out the questionnaire. Participants who met the pre-screening criteria were invited via Prolific to complete the survey online on Qualtrics three times at 4-month intervals (Time 1 was open 20 April 2017, Time 2 6 November 2017, and Time 3 7 March 2018). Nine hundred eighty-two individuals started the study at T1 and 565 at T3. All participants were paid £3 for the study. Attention check questions were inserted in the survey to ensure participant attention. The sample to be used in this study included only participants who passed the attention test.

#### 3.1.3. Measures

In addition to sociodemographic questions (age, country of residence, gender, education, and work status), we measured the Balance, job burnout, overall job satisfaction, job turnover intention, counterproductive behaviors at work, problematic alcohol use, disordered sleep and somatic complaints. All measures were completed three times at 4-month intervals. We used a forced-choice format in Qualtrics to prevent missing data. Reliability for all measures was estimated using Cronbach’s alpha and is presented in Table 4. All measures had good internal consistency.

*Job burnout* was measured like in Study 1. The Balance was composed of 39 bipolar items rated on a 11-point scale (from −5 to +5, including 0). In addition to the 34 valid items that were identified at phase 1, we added 4 new items to the Balance to give more weight to workload and 1 new item for the meaning of work. The global score was computed by summing up the items. Positive scores indicate that the respondent has more (or heavier) resources than risks and negative scores indicate that the respondent has more (or heavier) risks than resources. A zero score means that the respondent has the same level of risks and resources.

*Job turnover* intention was assessed by averaging three items: “I often think about quitting my company”, “I intend to search for a position with another employer within the next year”, “I intend to leave my company in a near future” [33]. Respondents indicated their level of agreement with each item using an 8-point Likert scale (1 = never or less than once a year to 8 = a few times a day).

*Overall job satisfaction* was measured by averaging three items from the Job Satisfaction Index [37]. The items retained were: “All in all, I’m very satisfied with my current job”, “In general, my job measures up to the sort of job I wanted when I took it”, “Knowing what I know now, if I had to decide all over again whether to take my job, I would”. Respondents indicated their level of agreement with each item using a 7-point Likert scale (1 = strongly disagree to 7 = strongly agree).

*Counterproductive behavior at work* was assessed using a selection of seven items from the counterproductive work behavior checklist (CWB-C) [38]. Respondents indicated their level of agreement with the following items: “At work, I take longer breaks than I should”; “I purposely work slowly when things need to get done”; “At work, I purposely fail to follow instructions”; “I tell people outside the job what a lousy place I work for”; “I’m nasty or rude to a client or a colleague”; “I have bursts of anger at work (e.g., I scream and/or throw objects,…)”; “I steal something belonging to my company”, using an 8-point Likert scale (1 = never or less than once a year to 8 = a few times a day). A global score was obtained by averaging the item scores.

*Depression* was measured by the PHQ−8 (depression module of the ‘‘Patient Health Questionnaire [39]). Participants indicated how often they experienced problems such as “poor appetite or overeating”, using a 4-point Likert scale, ranging from 1 = Not at all to 4 = nearly every day. A global score was obtained by summing up the scores.

*Problematic alcohol use* was measured using the two “problematic alcohol use” items from the Comprehensive Inventory of Substance and Behavioral Addictions (CISBA) [40]: “During the last three months, has your alcohol consumption brought about negative consequences in your everyday life (e.g., reproaches from or quarrels with the family and/or friends, judicial problems, health problems, negative impact on professional life)?”, and “During the last three months, it has been difficult for me to refrain from drinking”, both rated on a four-point Likert scale, from fully disagree to fully agree. A global score was obtained by averaging the item scores.

*Disordered sleep* was evaluated by a short questionnaire assessing frequency of sleep difficulties (sleep onset latency > 30 min; nocturnal awakenings > 30 min; waking > 20 min before alarm; nightmares) and subjective sleep quality during the last month on a four-point scale (never; less than once a week; once or twice a week; three times a week or more). A score for sleep problems was obtained by averaging the item scores.

*Somatic complaints* were assessed using a selection of the ten most frequent symptoms of the Pennebaker Inventory of Limbic Languidness (PILL) [41], a widely used symptom checklist of common physical symptoms (backache, headache, stomachache, running nose,…). Participants were asked to indicate how frequently they experienced each item (e.g., headache or migraine), on an 8-point Likert scale (1 = never or less than once a year to 8 = a few times a day). A global score was obtained by averaging the item scores. Reliabilities were not computed as symptoms are not expected to covary (i.e., a person who has frequent migraine is not necessarily expected to have frequent backache).

### 3.2. Data Analysis

Preliminary analyses involved computing means and reliabilities for each variable at each time. To address the first research question, we computed linear correlations among variables, both cross-sectionally and prospectively. Then, to allow readers to visualize more easily what happens to the criterion variables when the Balance falls below zero, we represented the correlations between the Balance and criterion variables under the form of graphs. Because correlations coefficients were very similar within and across time, we did it for Time 1 only in order not to lengthen the paper unnecessarily.

To address the second research question and simultaneously consider intra- and inter-individual changes over time, we ran a hierarchical linear model (HLM). Indeed, HLM allows to include predictors measured at different levels (i.e., Level 1, within-individual across time; Level 2, between individuals), and thereby yield better estimates of standard error and effects [42]. The analysis of change was conducted using a multilevel modeling (MLM) framework with the HLM 7.03 software. MLM estimates are based on all the available data at Level 1, but without imputing data. We used maximum likelihood estimation, which does not require the assumption of missingness completely at random.

We first estimated an unconditional means model for burnout, which did not include any predictors or control variables. The unconditional means model was only run to calculate the Intraclass Correlation Coefficient (ICC). The ICC–estimated by dividing the Level 2 variance by the total variance in a model with no predictors–allowed us to evaluate the relative magnitude of the within-person (Level 1) and the between-person (Level 2) variance components of job burnout. The ICC is also a measure of the average autocorrelation of the dependent variable over time, giving an index of the average stability of job burnout. In the next step, we calculated the unconditional growth model where time is the only predictor, to examine the patterns of change in job burnout over time.

Subsequently, we tested the conditional model. MLM allows both time-varying and time-invariant predictors to be included in the models. As a result, we were able to predict the change in job burnout from changes in the balance as well as from the time-invariant predictors (gender, age, and work status). The time-varying predictor was added to the level 1 equation, and the three time-invariant predictors to the level 2 equation. At level 1, the time-varying predictor was within-person centered in order to address bias due to unobserved heterogeneity or unmeasured factors that vary across individuals and have a consistent effect over time on the construct of interest [43]. It was also constrained to have fixed effects. Its average level over the three assessment waves was calculated and added as predictor of the slope coefficient at level 2. This procedure aimed to examine the pure effect of change in the time-varying predictor (i.e., the balance) over time (as its mean level was controlled [44]).

### 3.3. Results

The means, standard deviations, ranges, and reliabilities of all the variables are presented in the (Supplemental Material Table S1).

#### 3.3.1. Relations between the Balance and Criterion Variables

Linear correlations between the Balance and all variables under investigation at all measurement times are presented in the (Supplemental Material Table S2). The coefficients indicated coherent patterns of relations, which support the validity of the Balance. At Time 1, 2 and 3, the Balance was most strongly negatively correlated with burnout, followed by turnover intention, and was strongly positively correlated with job satisfaction (all with large effect-sizes, both cross-sectionally and prospectively). In addition, higher scores on the Balance were associated with less somatic complaints, counterproductive behavior, disordered sleep (all with medium effect-sizes) as well as with less problematic alcohol use (small effect-size).

#### 3.3.2. Changes in the Balance as a Predictor of Changes in Job Burnout

Estimated from the unconditional means models, the ICC was 0.79, suggesting that the variance in job burnout was largely due to differences between participants and that job burnout was almost stable (average stability 0.79). Results from the unconditional growth model displayed a negative slope (i.e., association with time) value meaning that job burnout decreased by 0.29 points per wave. The results from the random sections indicated significant individual variability around the slope (10.42, *p* < 0.001) and showed that it was appropriate to examine the predictors of the rates of job burnout linear change.

The results of the conditional model are presented in Table 4. Changes in the Balance predicted changes in job burnout (i.e., the strength of the linear association between time and job burnout). For every unit increase in the Balance (i.e., every unit deviation from the person-specific mean) over a wave, a decrease of 0.06 units of job burnout was evident. The average level of the balance also predicted changes in job burnout. For every unit above the average level of the balance, a decrease of 0.001 units of support and of 0.07 units of job burnout were evident over a wave. Turning to other time-invariant predictors, age and work status had no significant relations to job burnout but gender did. Compared to men, women displayed an increase of 0.76 units of job burnout over a wave. The variance components listed in the bottom section of Table 4 reflect the proportion of variance in job burnout on each level of analysis that was not explained by the effects described above. Variance components on Levels 1 and 2 indicated that a significant portion of the variance remains unexplained.

## 4. Discussion

The aim of the present study was to develop a valid and conceptually reliable instrument that we called the *Balance*, to assess job demands and resources, based on the theoretical framework of JD-R. In Study 1, we checked how the Balance was related to job burnout, subjective health and turnover intention. In Study 2, we looked at the longitudinal relations that exist between the Balance and job burnout, overall job satisfaction, job turnover intention, counterproductive behaviors at work, depression, alcohol use, sleep disorders and somatic complaints.

The results point to the important relation there is between the Balance and job burnout. First, the Balance score and the MBI were highly correlated. As expected, participants whose balance leaned to the positive side, meaning that they had more resources than risk factors, had significantly lower scores on MBI and participants whose balance leaned to the negative side, meaning they had more risk factors than resources, had a significantly higher score on MBI. This relation held both longitudinally and cross-sectionally and across different samples. Importantly, in Study 1, after controlling for demographic factors, the Balance alone accounted for 47% of the variance in job burnout scores. In Study 2, results indicated that a change in the Balance produced significant change in burnout scores over time. Therefore, the Balance appears to be a sensitive tool to predict change in job burnout. In other words, based on the Balance scores of employees, we can predict the course of their burnout scores and understand how steep the change in their job burnout scores may be. As explained later in the discussion, this bears very practical implications for companies.

The correlation between the Balance and other constructs also deserves consideration. The Balance was positively related to positive outcomes (i.e., overall job satisfaction and subjective health) and negatively related to negative outcomes (i.e., job turnover intention, counterproductive behaviors at work, depression, alcohol use, sleep disorders and somatic complaints). These associations were in the expected direction and consistent with the literature (e.g., [20,23,24]). It is interesting to note that the Balance did not only relate to job-related outcomes such as turnover intention or job satisfaction but also to a broad set of psychological indicators of stress/strain such as depression, problematic alcohol use, disordered sleep and somatic complaints. Although more rigorous research about the relation between the Balance score and clinical disorders is needed (e.g., interventional studies), these findings constitute additional evidence in favor of the validity of the instrument.

Given the wide application of the JD-R model and job burnout, we believe the generic nature of the Balance instrument holds considerable potential for clinicians, companies and researchers interested in assessing job demands and resources. In clinical practice with burned out patients, the Balance can be used during intake assessment in order to identify the heavier risk factors and the absent resources, thereby providing useful clinical directions. The Balance can also be used as a diagnostic tool by companies of all sizes to identify organizational risk factors (e.g., lack of support by supervisors) as well as employees whose balance is null or leans on the wrong side, which allows to take actions toward burnout prevention. From a research perspective, the Balance is an original tool that combines multiple risk factors with protective factors to form a single instrument.

The current research has some limitations. We had a Western and generally educated sample that had access to numeric facilities. Although the Balance is meant to be used for employees in different occupational categories from different educational backgrounds, additional validation is warranted, especially for non-Western populations and blue-collar workers, such as factory workers that may suffer from the digital divide. In Study 2, there was attrition from waves 1 to 3, which resulted in missing data. Particularly lower rates of participation from older participants constrained the current study and the overall sample size. In addition, we did not measure how the Balance was related to job engagement. Given the link between job resources and job engagement, it could be interesting to see whether participants whose balance leaned towards the positive side also had higher job engagement scores [20]. Future studies could therefore benefit from using a more diverse sample and looking in the associations between job engagement and the Balance.

## 5. Conclusions

In conclusion, our findings point to the utility of an instrument that includes both risk and resources as bi-polar items, which can overcome shortcomings of using multiple measures of risks and resources. What distinguishes the Balance from other measures is its consistent patterns of association with a wide range of constructs (job burnout, somatic symptoms, turnover intention) and across diverse populations (French-speaking and English-speaking populations) and over time. While additional studies are warranted especially for employees with lower education levels such as blue-collar workers, and non-western populations the present study contributes to the literature by offering a measure that predicts burnout and a canvas for measuring the balance between risks and resources. It is our hope that the instrument will continue to evolve based on future research findings in order to further increase its explanatory power.

## Figures and Tables

**Table 1:** Mean values, standard deviations, range and intercorrelations of variables.

	*N*	Mean	*SD*	Possible Range	Reliability	1	2	3	4
The Balance	656	42.11	48.53	−175 to +175	N/A	1	−0.69 **	0.33 **	−0.53 **
Job burnout	656	1.75	1.07	0 to 6	0.88		1	−0.39 **	0.56 **
Subjective Health	610	4.73	1.15	1 to 7	0.78			1	−0.21 **
Turnover Intention	656	1.97	1.89	0 to 6	0.88				1

** *p* < 0.01. The Balance = Balance between risks and resources (positive scores indicate more resources than risks). N/A = Not Applicable: Internal consistencies were not computed for these scores, as responses to the items were not expected to be consistent with each other.

**Table 2:** Descriptive statistics of items and their correlations with the MBI.

Item No	Risk Factor	Mean	*SD*	*r*
1	Work/life conflict	0.90	2.81	0.36 **
2	Stressors on the road to work	0.79	3.24	0.19 **
3	Work load	−1.67	2.17	−0.17 **
4	Time pressure	−0.15	3.01	0.11 **
5	Lack of rewards	−0.21	3.06	0.51 **
6	Role conflict	0.84	2.84	0.45 **
7	Role ambiguity	2.37	2.56	0.40 **
8	Lack of variety	2.62	2.48	0.41 **
9	Lack of autonomy	2.53	2.39	0.40 **
10	External control	1.96	2.43	0.34 **
11	IT complications	1.73	2.38	0.25 **
12	Interruption of work	−0.99	2.73	0.17 **
13	Hiding feelings	0.29	2.93	0.53 **
14	Showing unfelt emotions	0.85	2.77	0.35 **
15	Negative affect	0.40	2.74	0.31 **
16	Lack of positive affect	2.41	2.03	0.25 **
17	Introversion	1.85	2.44	0.28 **
18	Pessimism	1.70	2.44	0.35 **
19	Perfectionism	0.71	2.79	0.40 **
20	Lack of assertiveness	1.15	2.69	0.33 **
21	Difficulties in Emotion management	1.62	2.20	0.28 **
22	Conflict of values	0.85	2.97	0.54 **
23	Uncaring company	0.19	3.18	0.54 **
24	Lack of justice	0.39	2.69	0.53 **
25	Health risks	2.53	2.91	0.33 **
26	Bullying or harassment	2.55	2.97	0.45 **
27	Supervisor selfish	1.00	3.25	0.48 **
28	Supervisor not supporting	1.73	2.77	0.49 **
29	Supervisor not motivating	0.89	3.14	0.51 **
30	Lack of recognition by the supervisor	1.27	3.10	0.49 **
31	Refused (vacation) leave	3.33	2.23	0.26 **
32	Uncomfortable work schedule	2.08	2.73	0.26 **
33	Unsupportive colleagues	2.34	2.40	0.35 **
34	Bad atmosphere on the workplace	1.73	2.76	0.49 **
35	Stress due to colleagues	1.71	2.62	0.43 **

** *p* < 0.01.

**Table 3:** Regression coefficients for models testing the effects of demographic variables of age, gender, work status and risk and protective factors on participant’s job burnout scores.

		Model 1				Model 2 ^+^		
	*B*	*SE*	Beta	*t*	*B*	*SE*	Beta	*t*
Intercept	30.99	3.58		8.67	46.19	2.64		17.48
Female	4.62 **	1.43	0.13 **	3.23	2.12 *	1.03	0.06 *	2.05
Age	−0.15 *	0.07	−0.09 *	−2.27	−0.18 ***	0.05	−0.11 ***	−3.81
Work status	−0.03	1.92	−0.001	−0.02	−2.52	1.38	−0.05	−1.82
The Balance					−0.24 ***	0.01	−0.69 **	−24.73
*R^2^*	0.03 ***				0.50 ***			
*ΔR^2^*					0.47 **			

The Balance = Balance between risks and resources (positive scores indicate more resources than risks. * *p* < 0.05; ** *p* < 0.01; *** *p* < 0.001. ^+^, Compared with Model 1.

**Table 4:** Results of the HLM conditional models of the balance of risk and resources, gender, age and work status predicting change in job burnout.

Fixed Effects	Coefficient (*SE*)	*t*	*df*
Intercept (level of job burnout at time 1)	37.14 *** (0.71)	52.54	879
Level 1 time-varying covariate			
Balance of risk and resources	−0.06 *** (0.00)	−8.60	180
Level 2 time-invariant covariates			
Intercept (mean growth rate)	−0.95 (0.64)	−1.48	875
Balance of risk and resources (Mean)	−0.07 *** (0.00)	−20.17	875
Gender	0.76 * (0.35)	2.15	875
Age	−0.03 (0.02)	−1.22	875
Work status	−0.18 (0.40)	−0.46	875
Random effects	Variance (*SD*)		
Intercept, r_o_	267.24 *** (16.35)		
Wave, r_1_	19.40 *** (4.40)		
Level-1, *e*	52.41 (7.24)		

* *p* < 0.05; *** *p* < 0.001.

## Data Availability

Publicly available datasets were analyzed in these studies. This data can be found here: (https://osf.io/wq37s/?view_only=7644fc1a3af5447195163bf50f4a85ce accessed on 5 July 2021).

## Supplementary Materials

The following are available online at https://www.mdpi.com/article/10.3390/ijerph18179416/s1, Table S1: Means, standard deviations and internal consistencies (Cronbach’s alpha) for all variables under investigation at all measurement times, Table S2: Correlations between predictor and outcome variables.
