# Supplementary material for: An Instrument to Operationalize the Balance between Risks and Resources and Predict Job Burnout
Source: Int J Environ Res Public Health. 2021 Sep 6;18(17):9416. doi: 10.3390/ijerph18179416 (PMC8431336; doi:10.3390/ijerph18179416)
Supplement: Supplementary file 1 [file ijerph-18-09416-s001.zip › ijerph-1308070-supplementary.pdf]

An Instrument to Operationalize the Balance between Risks and Resources and Predict Job Burnout

Neda Bebiroglu, Marie Bayot, Benjamin Brion, Léopold Denis, Thomas Pirsoul, Isabelle Roskam, and Moïra Mikolajczak

Supplemental material

Table S1

Means, standard deviations and internal consistencies (Cronbach's alpha) for all variables under investigation at all measurement times

	Time 1				Time 2			Time 3		
	Possible range	Mean	SD	Reliability	Mean	SD	Reliability	Mean	SD	Reliability
The Balance	-195+195	35.82	62.08	N/A	39.57	61.1	N/A	42.95	61.42	N/A
Burnout	0-96	37.26	17.48	.89	37.01	17.76	.90	36.39	18.03	.90
Turnover intention	1-8	2.88	2.16	.95	2.76	2.03	.94	2.82	2.14	.96
Job satisfaction	1-7	4.88	1.66	.91	4.91	1.64	.93	4.93	1.64	.93
Counterproductive beh	1-8	1.81	1.03	.83	1.70	.82	.75	1.74	.95	.80
Depression	8-32	14.24	5.35	.90	14.18	5.48	.91	13.81	5.32	.90
Sleep disorders	1-4	2.29	.75	.80	2.23	.74	.79	2.21	.75	.82
Alcohol consumption	1-4	1.25	.59	.80	1.23	.56	.82	1.23	.56	.79

Somatic complaints	1-8	2.74	1.26	N/A	2.65	1.22	N/A	2.64	1.21	N/A

N/A = Not Applicable: Internal consistencies were not computed for these scores, as responses to the items were not expected to be consistent with each other (for instance, a person having frequent migraines is not necessarily expected to have frequent backache).

Table S2

Correlations between predictor and outcome variables

	1	2	3	4	5	6	7	8	9	10	11	12	13	14	15
1. W1_Balance	1											.			
2. W2_Balance	.60**	1													
3. W3_Balance	.61**	.68**	1												
4. W1_Burnout	-.62**	-.55**	-.54**	1											
5. W2_Burnout	-.56**	-.63**	-.60**	.80**	1										
6. W3_Burnout	-.54**	-.59**	-.64**	.70**	.83**	1									
7. W1_Turnover Intention	-.48**	-.44**	-.44**	.66**	.56**	.57**	1								
8. W2_Turnover Intention	-.46**	-.49**	-.50**	.59**	.65**	.58**	.73**	1							
9. W3_Turnover Intention	-.46**	-.52**	-.55**	.56**	.60**	.68**	.69**	.77**	1						
10. W1_Job Satisfaction	.58**	.55**	.52**	-.71**	-.66**	-.63**	-.69**	-.59**	-.60**	1					
11. W2_Job Satisfaction	.52**	.62**	.56**	-.61**	-.73**	-.67**	-.54**	-.68**	-.64**	.75**	1				
12. W3_Job Satisfaction	53**	.61**	.64**	-.63**	-.71**	-.75**	-.58**	-.70**	-.76**	.75**	.82**	1			
13. W1_Counterproductive	-.22**	-.25**	-.24**	.45**	.42**	.40**	.32**	.44**	.33**	-.27**	-.30**	-.30**	1		
14. W2_Counterproductive	-.24**	-.21**	-.31**	.38**	.45**	.37**	.36**	.40**	.40**	-.25**	-.31**	-.36**	.66**	1	
15. W3_Counterproductive	-.21**	-.26**	-.26**	.38**	.44**	.44**	.39**	.34**	.40**	-.29**	-.35**	-.36**	.71**	.67**	1
16. W1_Depression	-.40**	-.34**	-.36**	.52**	.49**	.53**	.39**	.34**	.39**	-.35**	-.31**	-.36**	.36**	.24**	.29**
17. W2_Depression	-.40**	-.45**	-.39**	.48**	.58**	.54**	.34**	.43**	.43**	-.35**	-.40**	-.42**	.31**	.33**	.35**

18. W3_Depression	-.34**	-.36**	-.39**	.44**	.50**	.56**	.33**	.32**	.43**	-.34**	-.34**	-.40**	.26**	.22**	.34**
19. W1_Alcohol Use	-.08*	-.03	-.08	.16**	.09*	.14**	.14**	.02	.13**	-.04	.00	-.04	.30**	.24**	.25**
20. W2_Alcohol Use	-.16**	-.08*	-.08	.11**	.11**	.12**	.09*	.06	.17**	-.03	-.01	-.08	.31**	.32**	.26**
21. W3_Alcohol Use	-.18**	-.06	-.08	.16**	.10*	.17**	.17**	.13**	.21**	-.10*	-.07	-.10*	.30**	.25**	.32**
22. W1_Sleep Disorders	-.27**	-.28**	-.22**	.31**	.34**	.33**	.25**	.23**	.23**	-.21**	-.24**	-.23**	.18**	.13**	.13**
23. W2_Sleep Disorders	-.27**	-.33**	-.25**	.29**	.37**	.35**	.17**	.25**	.22**	-.18**	-.26**	-.25**	.19**	.18**	.18**
24. W3_Sleep Disorders	-.23**	-.27**	-.20**	.27**	.31**	.34**	.17**	.14**	.23**	-.19**	-.20**	-.23**	.18**	.18**	.21**
25. W1_Somatic Complaints	-.23**	-.31**	-.24**	.31**	.35**	.36**	.26**	.26**	.26**	-.18**	-.24**	-.24**	.32**	.24**	.24**
26. W2_Somatic Complaints	-.23**	-.28**	-.21**	.30**	.36**	.33**	.17**	.24**	.23**	-.19**	-.24**	-.21**	.16**	.23**	.15**
27. W3_Somatic Complaints	-.21**	-.27**	-.22**	.26**	.30**	.33**	.17**	.19**	.26**	-.16**	-.20**	-.20**	.17**	.20**	.22**

Table S2

Continued

	16	17	18	19	20	21	22	23	24	25	26	27
1. W1_Balance												
2. W2_Balance												
3. W3_Balance												
4. W1_Burnout												
5. W2_Burnout												
6. W3_Burnout												
7. W1_Turnover Intention												
8. W2_Turnover Intention												
9. W3_Turnover Intention												
10. W1_Job Satisfaction												
11. W2_Job Satisfaction												
12. W3_Job Satisfaction												
13. W1_Counterproductive												
14. W2_Counterproductive												
15. W3_Counterproductive												
16. W1_Depression	1											
17. W2_Depression	.79**	1										

18. W3_Depression	.76**	.78**	1									
19. W1_Alcohol Use	.29**	.22**	.25**	1								
20. W2_Alcohol Use	.26**	.25**	.17**	.74**	1							
21. W3_Alcohol Use	.30**	.27**	.29**	.74**	.69**	1						
22. W1_Sleep Disorders	.58**	.51**	.48**	.19**	.16**	.16**	1					
23. W2_Sleep Disorders	.50**	.60**	.54**	.151	.18**	.16**	.76**	1				
24. W3_Sleep Disorders	.47**	.51**	.60**	.195	.17**	.21**	67**	.77**	1			
25. W1_Somatic Complaints	.54**	.50**	.50**	.214	.21**	.25**	.42**	.39**	.41**	1		
26. W2_Somatic Complaints	.52**	.54**	.46**	.134	.19**	.14**	.41**	.43**	.37**	.70**	1	
27. W3_Somatic Complaints	.49**	.49**	.55**	.247	.22**	.27**	.39**	.41**	.46**	.70**	.73**	1

**Correlation is significant at the 0.01 level (2-tailed).
